# Clinical significance of combined circulating *TERT* promoter mutations and miR-122 expression for screening HBV-related hepatocellular carcinoma

**DOI:** 10.1038/s41598-020-65213-8

**Published:** 2020-05-18

**Authors:** Ngo Tat Trung, Nghiem Xuan Hoan, Pham Quang Trung, Mai Thanh Binh, Hoang Van Tong, Nguyen Linh Toan, Mai Hong Bang, Le Huu Song

**Affiliations:** 1 Centre for Genetic Consultation and Cancer Screening, 108 Military Central Hospital, Hanoi, Vietnam; 2 Vietnamese-German Center of Excellence in Medical Research, Hanoi, Vietnam; 3 Institute of Clinical Infectious Diseases, 108 Military Central Hospital, Hanoi, Vietnam; 4 Department of Gastroenterology, 108 Military Central Hospital, Hanoi, Vietnam; 5 Institute of Biomedicine and Pharmacy, Vietnam Military Medical University, Hanoi, Vietnam; 6 Department of Pathophysiology, Vietnam Military Medical University, Hanoi, Vietnam

**Keywords:** Biological techniques, Biotechnology, Cancer, Genetics, Molecular biology

## Abstract

Telomerase reverse-transcriptase (TERT) gene promoter mutations in circulating cell-free DNA (cfDNA) as well as the levels of circulating microRNA-122 (miR-122) have been reported as potential noninvasive biomarkers for several. This study evaluates the diagnostic performance of potent biomarker-based panels composing of serological AFP, miR-122 and circulating *TERT* promoter mutations for screening HBV-related HCC. *TERT* promoter mutations (C228T and C250T) and miR-122 expression were assessed in the plasma samples from 249 patients with HBV-related liver diseases by nested PCR and qRT-PCR assays, respectively. The diagnostic values of *TERT* promoter mutations, miR-122 expression and biomarker-based panels were assessed by computation of the area under the curve (AUC). Nested-PCR assays were optimized to detect C228T and C250T mutations in TERT promoter with detection limit of 1%. The common hotspot C228T was observed in 22 HCC cases. The triple combinatory panel (AFP@TERT@miR-122) acquired the best diagnostic value to distinguish HCC from CHB (AUC = 0.98), LC (AUC = 0.88) or non-HCC (LC + CHB, AUC = 0.94) compared to the performance of double combinations or single biomarkers, respectively. Notably, among patients with AFP levels≤20 ng/μl, the double combination panel (TERT@miR-122) retains satisfactory diagnostic performance in discriminating HCC from the others (HCC vs. CHB, AUC = 0.96; HCC vs. LC, AUC = 0.88, HCC vs. non-HCC, AUC = 0.94). The triple combination panel AFP@TERT@miR-122 shows a better diagnostic performance for screening HCC in HBV patients, regardless of AFP levels. The newly established panels can be a potential application in clinical practice in Vietnamese setting.

## Introduction

The prevalence of the hepatitis B virus (HBV) infection and the incidence and mortality of HBV-related hepatocellular carcinoma (HCC) represent a serious health problem in most Asian countries including Vietnam^1^. The high mortality rate of HCC is due to the lack of suitable tools for early detection. So far, ultrasound and AFP (alpha-fetoprotein) are recommended for surveillance and early screening of HCC in high-risk groups^2,3^. However, roughly 30% of HCC patients had normal AFP levels at diagnosis even with advanced disease or high levels of AFP (>200 ng/ml) were also found in several non-HCC patients^4^. In addition, using ultrasound for HCC screening generates a poor sensitivity of 47%^5^ and the accuracy of ultrasound is highly dependent on the expertise and tumor size. The diagnostic performance of other serum protein biomarkers for routine surveillance of HCC is unsatisfactory^6^. Therefore, the implementation of potential biomarkers with better diagnostic performance and/or with the capability to complement current methods for early HCC detection in high-risk groups (e.g. HBV carriers) is urgently needed to improve the survival rate of HCC patients.

Recently, *TERT* promoter mutations have been described to stimulate the TERT transcription or telomerase activation in several types of cancers including HCC^7^. These mutations, located in two hotspots at 124 and 146 bases before the start codon ATG, create a new consensus binding site (CCGGAA or CCGGAT) for the transcription factors E-twenty-six (ETS) and Ternary Complex factor (TCF) increasing the activity of TERT promoter^8^. Previous studies have reported that *TERT* promoter mutations were detected in 59–68% of HCC tumor tissues^9,10^ and almost all *TERT* promoter mutations in HCC (95%) occurred at the first hot spot C228T (−124G > A)^10^. These findings revealed that *TERT* promoter mutations are the most frequent genetic alterations observed in HCC so far.

*TERT* promoter region is composed of high GC contents that make a technical challenge for designing clinically relevant assays to directly identify *TERT* promoter mutations from patients’ biopsies. So far, only one study reported a TaqMan real-time PCR for detecting *TERT* promoter mutations from tumor tissues^11^, but this assay did not show a technical detection limit, and difficult to be recapitulated^11^. Another study used Sanger sequencing for detecting *TERT* promoter mutations; it requires as abundance as at least 20% of mutant allele to establish a positive signal^12^. There are also additional studies using digital PCR to identify the prevance of Tert gene promoter mutations from blood of Spinal Myxopapillary Ependymoma^13^ or myxoid liposarcomas^14^ or metastatic melanoma patients^15^. However, the assay for direct identification of *TERT* promoter mutations from liquid biopsies in HCC have not been described, therefore the blood circulating prevalence of these mutations amongst malignant diseases like HCC has not been well addressed.

MicroRNAs (miRNA) are a class of small and endogenous non-coding RNA molecules known to post-transcriptionally modulate gene expression by negatively regulating the stability or translational efficiency of their target mRNAs. They are involved in controlling a wide array of biological processes such as cell proliferation, differentiation and apoptosis^16,17^. The aberrant expression of miRNAs was also documented in various malignant diseases including liver cancer^18–22^. The liver-specific miR-122 has been reported to play an important role in regulating hepatocytic differentiation, proliferation, maturation^23,24^, and carcinogenesis^20,21,25^. Previous studies have shown that the circulating levels of miR-122 in combination with AFP could be applied to improve the diagnostics of HCC in HBV patients^26–29^.

In this study, we evaluated a nested PCR assay for identification of *TERT* promoter mutations at technical detection limit in the range of 0.5–1% directly from peripheral blood. We evaluate the diagnostic performance of potent biomarkers-based panels (AFP, miR-122 expression and circulating *TERT* promoter mutations) for screening HBV-related HCC.

## Materials and Methods

All methods used in this study were in accordance with the relevant guidelines and regulations and were approved by the institutional review board and an independent Ethics Committee of the 108 Military Central Hospital, Hanoi, Vietnam.

### Patients and sampling

Two hundred forty-nine patients with HBV-related liver diseases were recruited for the study. These patients were treated in the 108 Military Central Hospital between 2016 and 2019. Patients were allocated into three groups including hepatocellular carcinoma (HCC, n = 96), liver cirrhosis (LC, n = 55) and chronic hepatitis (CHB, n = 98) based on the clinical manifestations, biochemical and liver function tests, imaging modalities (abdominal ultrasound, MRI or CT scanner). Histopathological analyses were mandatory for the diagnostic confirmation in case of HCC. All patients were negative for antibodies against HCV and HIV. Blood samples were obtained from all patients. Plasma was immediately separated from blood cells and were stored at −80 °C until use.

### Biochemical and serological tests

The levels of albumin, globulin, total and direct bilirubin, alanine transaminase (ALT), aspartate aminotransferase (AST) were measured on an auto-analyzer (Hitachi Automatic Analyzer, Tokyo, Japan). ALT and AST levels were assessed for both the patients and HCs. Markers for HBV infection (HBsAg, anti-HBc-IgM, anti-HBcIgG, HBeAg, and anti-HBe) were assessed by commercial immunoassay kits (General Biologicals Corp, Taipei, Taiwan and DiaSorin, Saluggia, Italy). AFP levels were measured using a commercial ELISA kit (General Biologicals Corp., Taipei, Taiwan) as previously described^29^.

### Circulating cell-free DNA extraction

Peripheral blood samples were spun at 1,600×g for 10 min at 4 °C. The plasma portion was re-centrifuged at 16,000×g for 10 min at 4 °C to obtain cell-free plasma and then were stored at −70 °C until further analyses. DNA was extracted from 4 mL of plasma using the QIAamp Circulating Nucleic Acid Kit (QIAGEN, Hilden, Germany) and following the manufacturer’s recommended protocol. The extracted circulating cell-free DNA (cfDNA) was then reconstituted in 100 μl of 25 mM Tris-HCl pH 8.0 containing 0.5 mM EDTA and stored at −20 °C for further analyses.

### Nested PCR assays for the identification of *TERT* promoter mutations

The promoter region of *TERT* gene (Accession number AH007699.2) was amplified in Eppendorf thermocycler using 5 μl of reconstituted cfDNA as template with the outer primer pairs: Tr-Tert-seq-F: 5'-GTC CTG CCC CTT CAC CTT CCA-3' and Tr-Tert-seq-R: 5'- GCA GCG CTG CCT GAA ACT CG-3' plus 0.2 unit of Promega Gold Tag DNA Polymerase (M3001), 8% DMSO and 1.25 M Betaine to generate an amplicon of 163 bp. The PCR condition was set with an initial denaturation at 95 °C for 5 min, followed by 30 cycles of 95 °C for 30 sec and 62 °C (annealing) for 45 sec and elongation at 72 °C for 30 sec. Afterwards, the outer amplicon products were then diluted 1000 times; 5 μl of diluted outer PCR product was used as a template for the nested PCRs.

For identification of *TERT* C228T and C250T mutations, two allele-specific PCRs with individual forward primers: Tr-ARMS-TERT-MT(C228T)-F2: 5'-CCC CGG CCC AGC CCGT-3' and Tr-ARMS-C250T-MT-F: 5'-CGC CCC GTC CCG ACC CCGTC-3' (the underlined letters are mismatched nucleotide to further eliminate the unspecific pairing of forward primers to wild-type templates) coupling to a single reverse primer (Tr-Tert-seq-R: 5'- GCA GCG CTG CCT GAA ACT CG-3') were used. Thermal cycling condition was the same as outer PCR to generate 93 bp amplicon (for C228T mutation) and 116 bp amplicon (for C250T mutation), respectively. The reaction mixtures were then electrophoresed against 2% to visualize the amplicon products (Supl. Figure 1).

### miRNA extraction and cDNA synthesis

Total RNA, including miRNA fractions, was isolated from 200 µl plasma with TRIzol reagent and reconstituted in 50 µl water treated with diethylpyrocarbonate (DEPC). The quality of total RNA preparations was assessed by NanoDrop spectrometer (NanoDrop Technologies, Wilmington, USA) at 260 and 280 nm (A260/280). Approximately 200 ng of total RNA were used for reverse transcription (RT) by RevertAid First Strand cDNA Synthesis Kit (ThermoFisher Scientific Inc, Singapore) following the manufacturer’s instruction. The specific primer used for miR-122 cDNA synthesis (miR-122 accession number: MIMAT0000421) was designed according to stem-loop theory as described previously^29,30^ (sequence: 5'-GTT GGC TCT GGT GCA GGG TCC GAG GTA TTC GCA CCA GAG CCA ACC AAA CA-3').

### Quantification of miRNA by real-time PCR

After reverse transcription, cDNA was reconstituted in 100 µl of 25 mM Tris-HCl pH 8.0. The real-time PCR (qPCR) reaction mixtures consisted of 10 µl of 2xSybr-Green I master mix (Applied Biosystems, Foster City, CA, USA), 5 μl of cDNA preparation, 5pmol of miRNA universal reverse primer 5'-GTG CAG GGT CCG AGG T-3' and 5 pmol of miR-122-specific forward primer (5'-GGT GTG GAG TGT GAC AAT GG-3'). The qPCR reaction was performed using Stratagene M3000p device (Stratagene, San Diego, CA, USA) with a pre-incubation step at 50 °C for 15 min, initial denaturation at 95 °C for 5 min, followed by 45 cycles of 95 °C for 15 sec and 60 °C for 60 sec. The RT-PCR reactions were finalised by amplicon melting dissociation. The cycle of threshold (Ct) values was recorded and analysed according to the comparative Ct method^31^, in which the Ct value of miRNA-16 was used as normalisation factor as recommended previously^29,32^.

### Statistical analysis

All statistical analyses were performed using R v3.2.2 (https://www.r-project.org/). Values were presented as either mean with standard deviation (SD), median with 25–75% percentiles, or numbers with percentages where appropriate. Chi-squared tests were used to test the significant differences of categorical variables between groups. Kruskal-Wallis or Mann Whitney Wilcoxon tests were used to compare non-parametric data of quantitative variables between different groups where appropriate. Logistic regression models were built to establish the potent biomarkers-based panels (AFP, miR-122 expression and circulating *TERT* promoter mutations) to predict HCC from individuals with chronic hepatitis B who did not have cancer at the time of the study. Receiver operating characteristic (ROC) curves were generated and the diagnostic value of different panels was assessed by computation of the area under the ROC curve (AUC). The level of significance was set at a two-sided *P* value of <0.05.

## Results

### Clinical characteristics of the patients

The baseline characteristics of the enrolled 249 HBV-infected patients are shown in Table 1. Most patients were male (84%). The median age of patients was higher in patients with later phase of liver disease progression. The levels of ALT were higher in patients with CHB compared to other subgroups (*P* < 0.0001). Total and direct bilirubin levels were significantly lower in patients with HCC compared to other subgroups (*P* < 0.0001). Albumin and prothrombin levels and blood cell counts were significantly lower in patients with LC compared to the other patient groups (*P* < 0.0001). AFP levels were significantly higher in HCC patients compared to CHB and LC groups (*P* < 0.0001) (Table 1).

**Table 1:** Characteristics HBV patients segregated according to clinical presentation.

Characteristics	CHB (n = 98)	LC (n = 55)	HCC (n = 96)	P value
Age (years)	42 [21–85]	57 [27–79]	55 [23–92]	<0.05^β^
Male (%)	81	78	91	NS^α^
AST (IU/L)	96 [35–293]	69 [50–134]	69 [48–123]	NS^β^
ALT (IU/L)	117 [36–453]	46 [29–85]	44 [30–84]	<0.0001^β^
Total bilirubin (mg/dl)	16 [12–61]	31 [22–58]	15 [11–20]	<0.0001^β^
Direct bilirubin (mg/dl)	5 [3–50]	15 [8–33]	4 [3–6]	<0.0001^β^
Albumin (g/L)	41 [38–43]	32 [28–38]	39 [35–42]	<0.0001^β^
Prothrombin (% of standard)	95 [81–108]	65 [53–79]	95 [85–106]	<0.0014^β^
WBC (x10^3^/ml)	7.0 [5.6–8.6]	5.0 [4.3–6.7]	7.0 [5.7–8.7]	<0.0001^β^
RBC (x10^6^/ml)	5.0 [4.6–5.3)	4.0 [3.4–4.2]	5.0 [4.3–5.2)	<0.0001^β^
PLT (x10^3^/ml)	191 [153–237]	88 [64–113]	194 [142–238]	<0.0001^β^
HBV-DNA - log10(copies/ml)	6.0 [4.4–8.0]	5.2 [3.9–7.8]	5.0 [4.2–6.6]	NS ^β^
AFP (IU/L)	2.0 [1.5–9.3]	11.0 [4.0–25.8]	126.0 [12.9–315.2]	<0.0001^β^

CHB, chronic hepatitis B; LC, liver cirrhosis; HCC, hepatocellular carcinoma; RBC, red blood cells; WBC, white blood cells; PLT, platelets. AST and ALT, aspartate and alanine amino transferase; AFP, alpha-fetoprotein; IU, international unit; NR, normal range. Values given are medians with 25–75% percentiles. P values were calculated by chi-squared (α) or Kruskal-Wallis test (β).

### Expression of miR-122 in HBV patients

**Figure 1:**
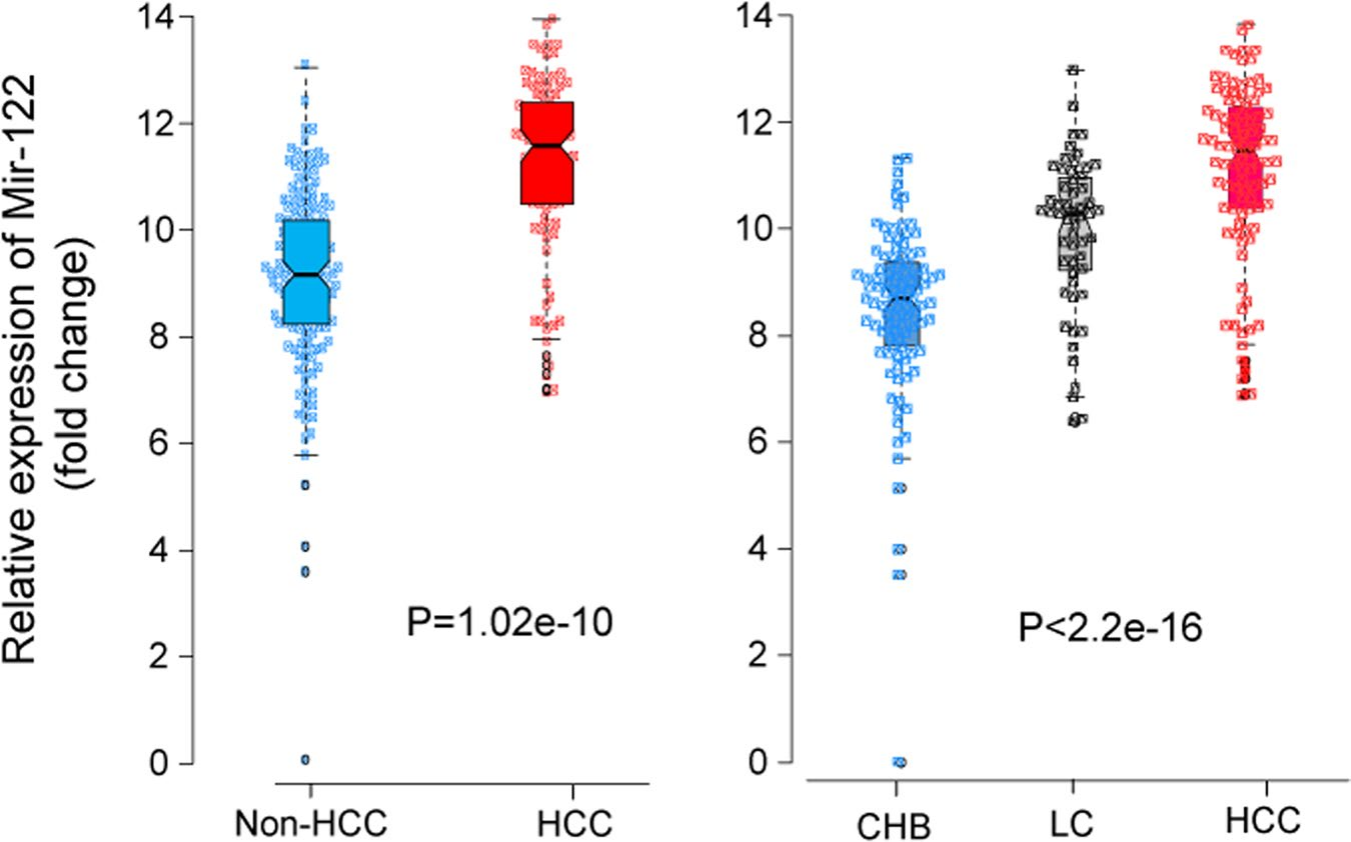
Relative expression of miR-122 in HBV patients. Differential expression of miR-122 in different groups of HBV related liver diseases including hepatocellular carcinoma (HCC), liver cirrhosis (LC), chronic hepatitis B (CHB). P values were calculated by Wilcoxon or Kruskal-Wallis test.

We assessed and compared the relative expression of miR-122 in different HBV patient subgroups. The results show that miR-122 relative expression was significantly higher in HCC patients compared to non-HCC patients (CHB + LC) (P < 0.0001). Relative expression of miR-122 was highest in HCC patients followed by LC and CHB patients indicating that miR-122 expression is increased according to the development of HBV-related liver diseases (Fig. 1).

### Circulating *TERT* promoter mutations in HBV related liver diseases

In order to determine the concentration of spiked *TERT* C228T and *TERT* C250T mutant alleles at which the nested PCR assays can detect, the genomic DNA of HCC cell lines HUH7^33^ (C228T carrier) and Mahlavu (C250 carrier)^33^ were mixed against genomic DNA myeloid leukaemia cell line HL-60 (unknown *TERT* promoter mutation) to make serial dilutions of 50%, 25%, 10%, 1%, 0.5%, 0.1% and 0% of either *TERT* C228T or *TERT* C250T mutations. All the dilutions were formulated to 20 ng/μl and we used 5ul (equal to 100 ng) as a template for nested PCRs. The nested PCR for the identifications of *TERT* C228T and *TERT* C250T acquired detection limits of 1% and 0.5% respectively (Supl. Figure 1). We then applied these allele-specific nested PCR assays onto the plasma samples collected from 249 patients with HBV-related liver diseases (96 HCC, 55 LC and 98 CHB). *TERT* C228T mutation was detected from 22 HCC plasma samples. However, we did not detect any *TERT* promoter mutations in CHB and LC subgroups and the C250T mutation was also not identified in our current study cohort.

### Diagnostic performance of the biomarkers and combined panels in differentiating HCC

**Figure 2:**
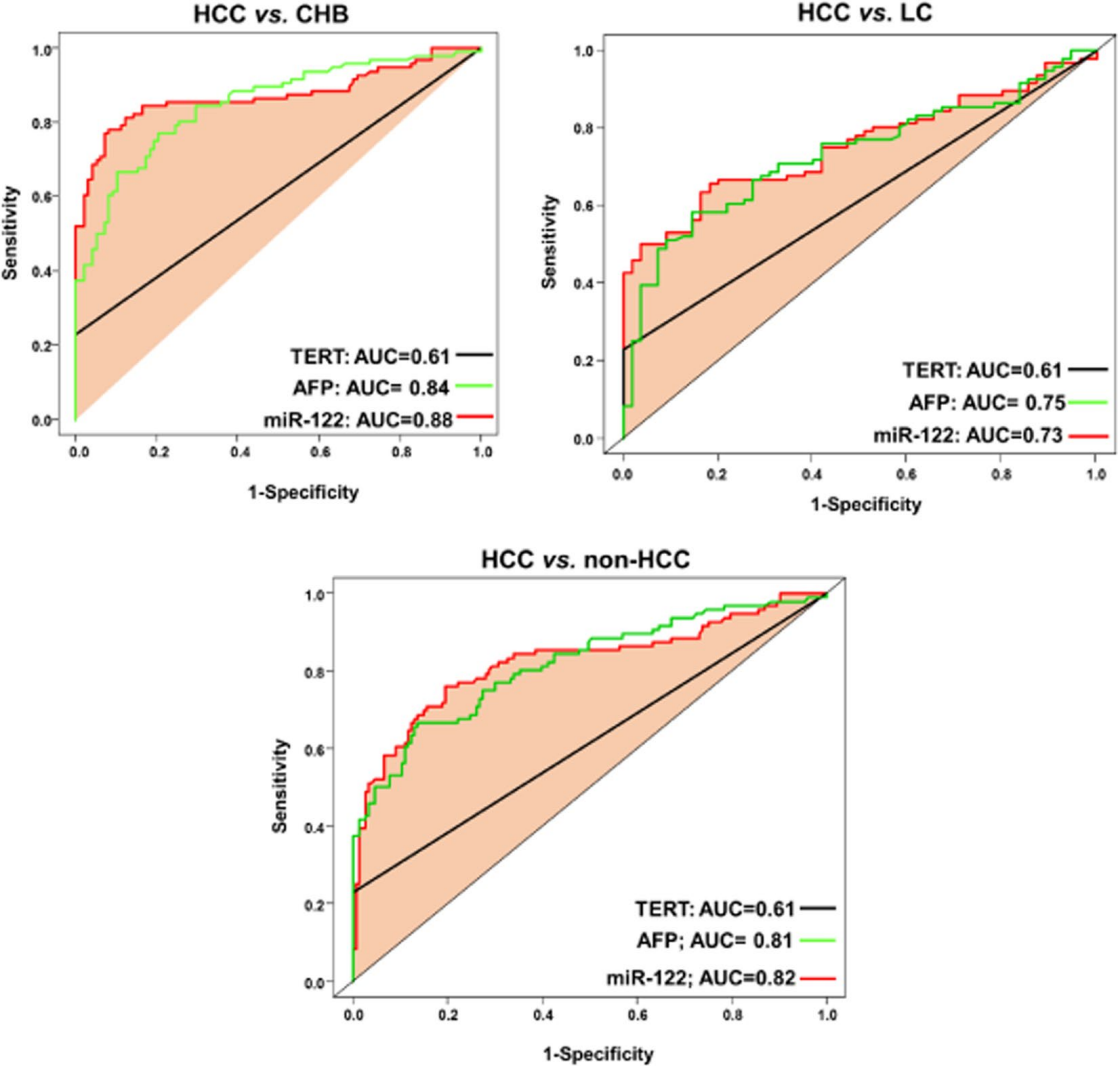
Diagnostic performance of single biomarker in differentiating HCC from other groups: HCC vs. CHB; HCC vs. LC; HCC vs. non-HCC.

**Figure 3:**
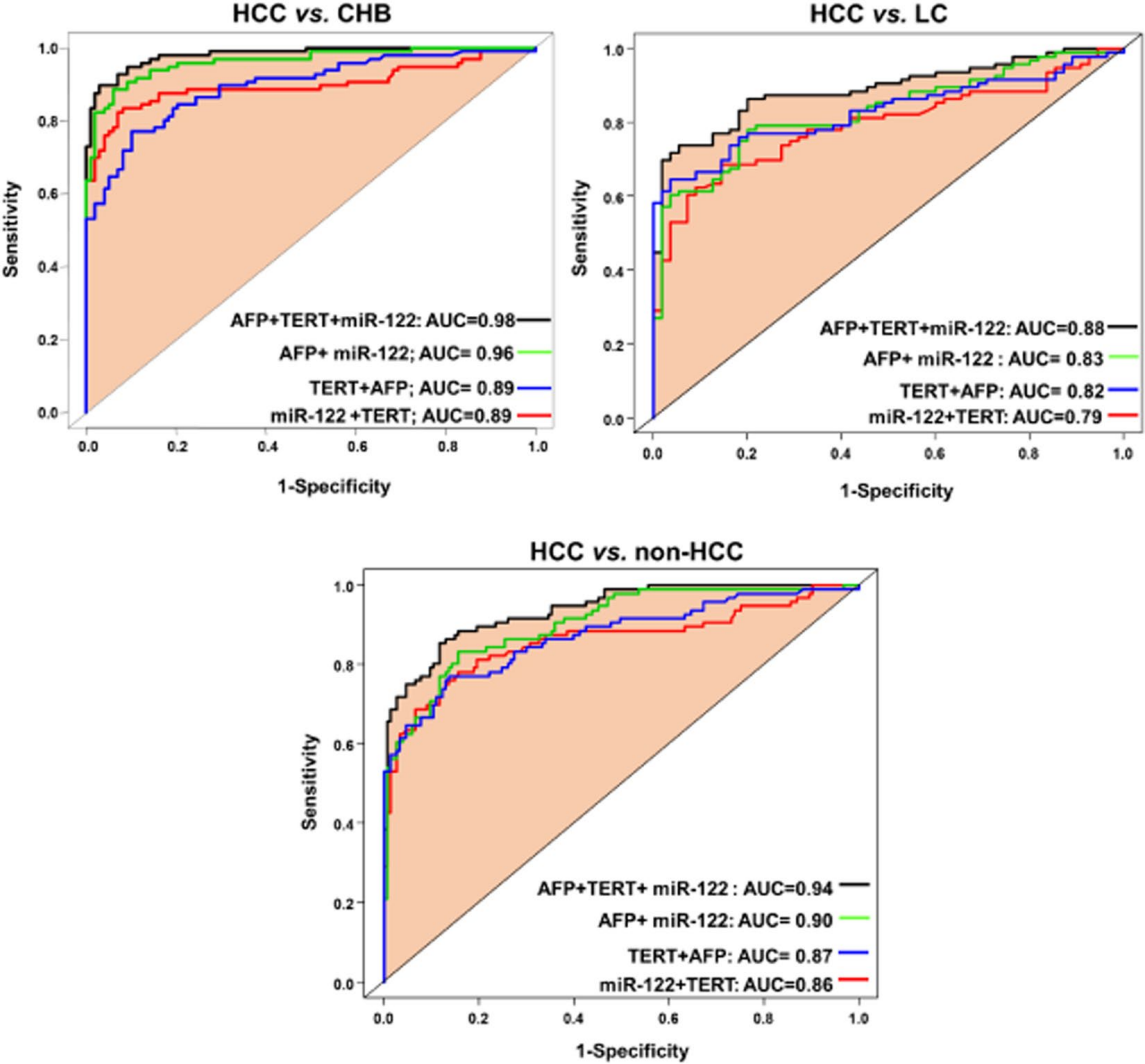
Diagnostic performance of the models with combination of the biomarkers (double or triple combination) in differentiating HCC from other groups: HCC vs. CHB; HCC vs. LC; HCC vs. non-HCC.

**Figure 4:**
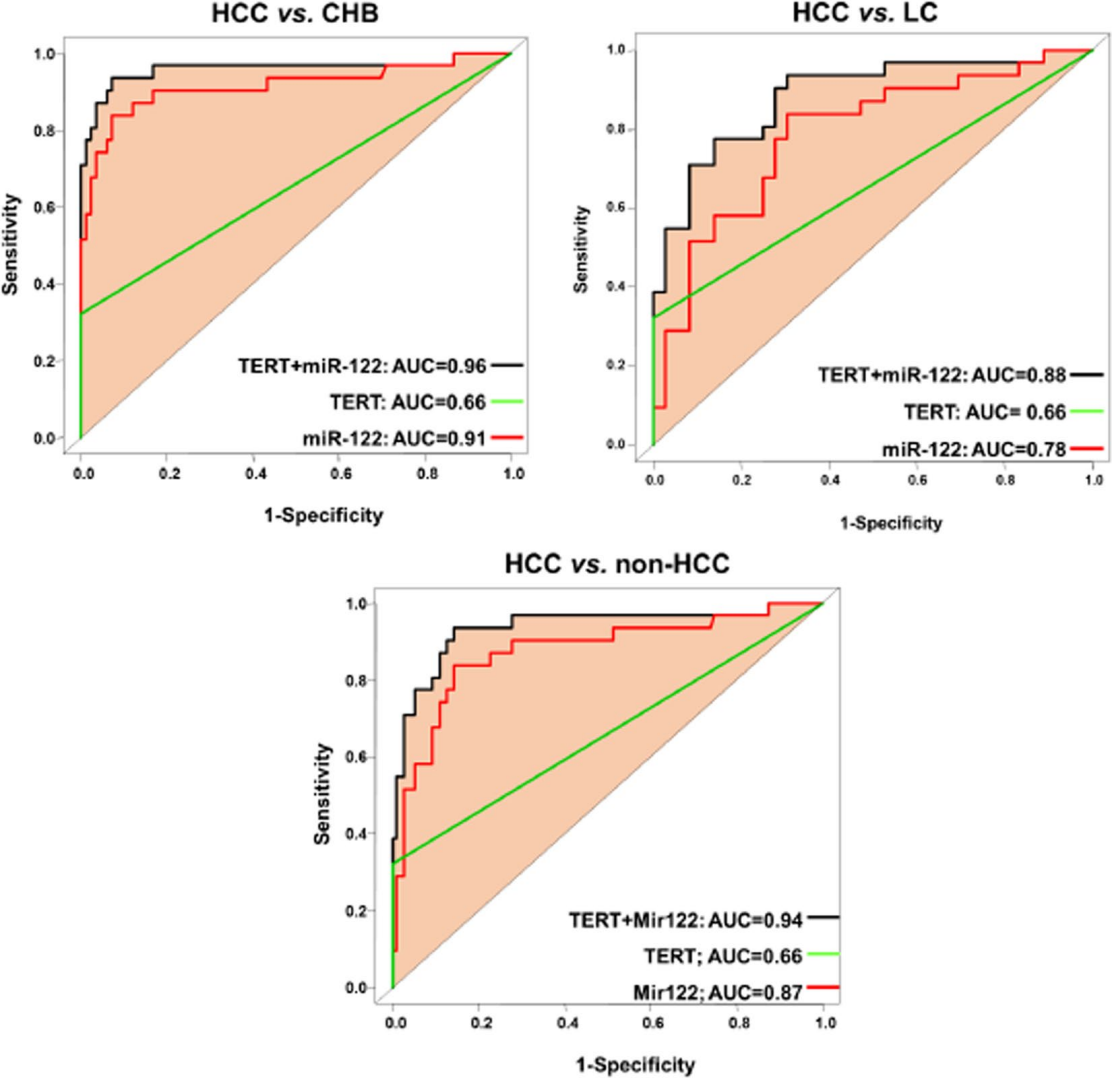
Diagnostic performance of the models with single or double combination of TERT promoter mutation and miR-122 expression in differentiating HCC from other groups at low AFP levels: HCC vs. non-HCC; HCC vs. LC; HCC vs. CHB.

We built logistic regression models to assess the diagnostic performance of the study biomarkers and different biomarkers-combinations (namely panels) for screening HCC. Single biomarkers: miR-122 expression, AFP levels and *TERT* C228T mutation) showed a poor or moderate diagnostic performance in differentiating HCC from LC (AUC = 0.73, 0.75, and 0.61, respectively), HCC from CHB (AUC = 0.88, 0.84, and 0.61, respectively) and HCC from non-HCC (AUC = 0.82, 0.81, and 0.61, respectively) (Fig. 2). Double combination of the biomarkers miR-122@AFP, AFP@TERT and TERT@miR-122 gained better diagnostic performance in differentiating HCC from LC (AUC = 0.83, 0.82, and 0.79, respectively), HCC from CHB (AUC = 0.96, 0.89 and 0.89, respectively) and HCC from non-HCC (AUC = 0.90, 0.87 and 0.86, respectively) (Fig. 3). Triple combination of the biomarkers (TERT@miR-122@AFP panel) showed the highest diagnostic performance in differentiating HCC from other forms of chronic liver diseases (HCC from LC, AUC = 0.88; HCC from CHB, AUC = 0.98; HCC from non-HCC or CHB + LC, AUC = 0.94) (Fig. 4). We also analyzed HCC diagnostic performance of single biomarkers and combined panels in HBV patients who had low AFP levels (≤20 ng/μl), the TERT@Mir122 panel still sustained its diagnostic accuracy in distinguishing HCC from the other groups [CHB patients, AUC = 0.96; LC patients AUC = 0.88, patients with non-HCC, AUC = 0.94] (Fig. 4).

## Discussion

The implementation of effective and reliable strategies to interrogate the risk populations such as HBV carriers to screen and detect HCC at early stage could improve the survival of suffered patients. Recently, a number of genetic lesions involving in key etiological pathways of HCC has been identified in patients’ liver tumor tissues^10,34–39^. Somatic mutations of *TERT* in the promoter region and circulating miR-122 have reported as potential noninvasive biomarkers for neoplastic diseases, including liver cancer^40,41^. In this study, we developed a PCR assay that could detect *TERT* promoter mutations from circulating DNA with high sensitivity and established the diagnostic panels with combinatory use of *TERT* promoter mutations, miR-122 expression and AFP levels for identifying HCC among chronic HBV-infected patients. The established models with the triple combination could provide a better diagnostic performance in the prediction of HCC occurrence among high-risk populations.

We used *TERT* promoter mutations and miR-122 in the combination with AFP in the predictive models based on the evidence on clinical relevance of these biomarkers in the association with liver cancer. MicroRNA (miRNAs) have been suggested as potential genetic biomarkers to predict tumor load, early disease recurrence and therapeutic response in various cancer entities, including HCC^20,22,26,42,43^. Among several important miRNAs, miR-122 is one of the most crucial microRNAs, which have been reported in liver cancer^40^. However, so far, only little is known about the values of miR-122 in diagnosis, treatment and prognosis of HCC even though miRNAs can be isolated from blood samples and easily integrated into standard follow up procedures. *TERT* promoter mutations are the most frequent somatic genetic alterations in HCC and *TERT* is the first recurrent gene somatically mutated in preneoplastic lesions in hepatocytes^10,33^. However, it is not clear how frequent these somatic lesions are detected in the blood of HCC patients. In fact, more than 90% of the region covering 1000 bp around *TERT* gene transcription start site is dominated by GC content, which makes a challenge for amplification of *TERT* promoter fragment especially from clinical samples containing a little amount of DNA. Therefore, the method for identification of *TERT* promoter mutations directly from peripheral blood, as well as the prevalence of circulating *TERT* promoter mutations in HCC has yet been described. With the application of amplification refractory mutation system^44^, our optimized nested PCR assays for identifying *TERT* promoter mutations C228T (−124G ≥ A) and C250T (−146G ≥ A) could detect *TERT* promoter mutation at a limit of 0.1–1%. Therefore, for the first time in this study, the prevalence of *TERT* gene promoter mutations circulating in blood was reported.

In our study cohort, *TERT* promoter mutations C228T (−124G ≥ A) and C250T (−146G ≥ A) were not detected in CHB and LC samples, whereas C228T (−124G ≥ A) mutation was detected in 23% of HCC samples. This prevalence is lower than that detected in liver tumor tissues as described previously^10^. Due to the low prevalence of C228T (−124G ≥ A) mutation in HCC plasma samples in our study compared to tumor tissues^9,10^, the single use of circulating C228T (−124G ≥ A) mutation acquires unsatisfactory diagnostics performance in assisting diagnosis of HCC. In addition, the diagnostic performance of either miR-122 expression or AFP levels alone did not gain enough accuracy, especially in discriminating HCC from LC. Nevertheless, the combinatory use of C228T (−124G ≥ A) mutation with two biomarkers miR-122 expression and AFP levels acquired a good diagnostic performance in distinguishing HCC from LC (AUC = 0.88) or from the group without HCC (LC + CHB, AUC = 0.94). Importantly, patients who have AFP levels lower than 20 ng/μl, the miR-122@TERT panel also provides accuracy in discriminating HCC from the other groups (CHB, AUC = 0.96; LC, AUC = 0.88, CHB + LC, AUC = 0.94). This result indicates that *TERT* promoter mutations could be an additional genetic marker for diagnostics of HCC in the circumstances of risk group and/or other biomarkers lacking accurate diagnostic performance.

There are some limitations remaining in our current study. Firstly, our assay could not be used for quantitatively monitoring the circulating *TERT* promoter mutations, therefore, it is impossible at the moment to use *TERT* promoter mutations for a treatment-follow-up purpose. Secondly, our study cohort was not big enough that allows us to investigate the association of *TERT* promoter mutations with liver function parameter and the progression of HCC. In conclusion, the combinatory use of *TERT* promoter mutation, miR-122 expression, together with serological AFP levels, is a potential biomarker assist the diagnostic establishment of HCC in HBV patients, particularly in HBV-related LC patients with normal AFP levels.

### Ethics approval and consent to participate

The study was approved by the institutional review board and an Independent Ethics Committee of the 108 Military Central Hospital, Hanoi, Vietnam. Informed written consent was obtained from all study patients.

## Supplementary information

Supplementary Information.

## Data Availability

Data and supporting materials associated with this study will be shared upon request.

## References

[1] WHO Representative Office, Vietnam, 2018; Available from, http://www.wpro.who.int/vietnam/topics/hepatitis/factsheet/en/

[2] Kudo M (2015). Clinical Practice Guidelines for Hepatocellular Carcinoma Differ between Japan, United States, and Europe. Liver Cancer.

[3] European Association for Study of, L., R. European Organisation for, and C. (2012). Treatment of, EASL-EORTC clinical practice guidelines: management of hepatocellular carcinoma. Eur. J. Cancer.

[4] Colombo M (2001). Screening for cancer in viral hepatitis. Clin. Liver Dis..

[5] Tzartzeva K (2018). Surveillance Imaging and Alpha Fetoprotein for Early Detection of Hepatocellular Carcinoma in Patients With Cirrhosis: A Meta-analysis. Gastroenterology.

[6] Marrero JA, Lok AS (2004). Newer markers for hepatocellular carcinoma. Gastroenterology.

[7] Huang DS (2015). Recurrent TERT promoter mutations identified in a large-scale study of multiple tumour types are associated with increased TERT expression and telomerase activation. Eur. J. Cancer.

[8] Bell RJ (2015). Cancer. The transcription factor GABP selectively binds and activates the mutant TERT promoter in cancer. Science.

[9] Nault JC (2014). Telomerase reverse transcriptase promoter mutation is an early somatic genetic alteration in the transformation of premalignant nodules in hepatocellular carcinoma on cirrhosis. Hepatology.

[10] Nault JC (2013). High frequency of telomerase reverse-transcriptase promoter somatic mutations in hepatocellular carcinoma and preneoplastic lesions. Nat. Commun..

[11] Remke M (2013). TERT promoter mutations are highly recurrent in SHH subgroup medulloblastoma. Acta Neuropathol..

[12] Normanno N (2009). Implications for KRAS status and EGFR-targeted therapies in metastatic CRC. Nat. Rev. Clin. Oncol..

[13] Deniel A (2019). TERTp Mutation Detection in Plasma by Droplet-Digital Polymerase Chain Reaction in Spinal Myxopapillary Ependymoma with Lung Metastases. World Neurosurg..

[14] Braig D (2019). Genotyping of circulating cell-free DNA enables noninvasive tumor detection in myxoid liposarcomas. Int. J. Cancer.

[15] Calapre L (2019). Locus-specific concordance of genomic alterations between tissue and plasma circulating tumor DNA in metastatic melanoma. Mol. Oncol..

[16] Davis-Dusenbery BN, Hata A (2010). MicroRNA in Cancer: The Involvement of Aberrant MicroRNA Biogenesis Regulatory Pathways. Genes. Cancer.

[17] Bartel DP (2009). MicroRNAs: target recognition and regulatory functions. Cell.

[18] Roderburg C, Luedde T (2014). Circulating microRNAs as markers of liver inflammation, fibrosis and cancer. J. Hepatol..

[19] Wang, K B. *et al*. Circulating microRNAs, potential biomarkers for drug-induced liver injury. *Proc. Natl. Acad. Sci. USA* 2009. 106. 10.1073/pnas.0813371106 PMC2657429 19246379

[20] Tang JC (2016). Circulating tumor DNA in hepatocellular carcinoma: trends and challenges. Cell Biosci..

[21] Schwarzenbach H (2014). Clinical relevance of circulating cell-free microRNAs in cancer. Nat. Rev. Clin. Oncol..

[22] YuqingHe JL (2015). Current State of Circulating MicroRNAs as Cancer Biomarkers. Clin. Chem..

[23] Davoodian N (2014). MicroRNA-122 overexpression promotes hepatic differentiation of human adipose tissue-derived stem cells. J. Cell Biochem..

[24] Shukla GC, Singh J, Barik S (2011). MicroRNAs: Processing, Maturation, Target Recognition and Regulatory Functions. Mol. Cell Pharmacol..

[25] Thakral S, Ghoshal K (2015). miR-122 is a unique molecule with great potential in diagnosis, prognosis of liver disease, and therapy both as miRNA mimic and antimir. Curr. Gene Ther..

[26] Lin XJ (2015). A serum microRNA classifier for early detection of hepatocellular carcinoma: a multicentre, retrospective, longitudinal biomarker identification study with a nested case-control study. Lancet Oncol..

[27] Zhou J (2011). Plasma microRNA panel to diagnose hepatitis B virus-related hepatocellular carcinoma. J. Clin. Oncol..

[28] Qi, P. *et al*. Serum microRNAs as biomarkers for hepatocellular carcinoma in Chinese patients with chronic hepatitis B virus infection. *Plos One*., **6**(12) (2011). 10.1371/journal.pone.0028486 PMC3234251 22174818

[29] Tat Trung N (2018). Optimisation of quantitative miRNA panels to consolidate the diagnostic surveillance of HBV-related hepatocellular carcinoma. PLoS One.

[30] Chen C (2005). Real-time quantification of microRNAs by stem-loop RT-PCR. Nucleic Acids Res..

[31] Livak KJ, S.T., Analysis of relative gene expression data using real-time quantitative PCR and the 2(-Delta Delta C(T)) Method. Methods., 2001. 25(4). 10.1006/meth.2001.1262 11846609

[32] Peltier HJ, Latham GJ (2008). Normalization of microRNA expression levels in quantitative RT-PCR assays: identification of suitable reference RNA targets in normal and cancerous human solid tissues. RNA.

[33] Cevik D, Yildiz G, Ozturk M (2015). Common telomerase reverse transcriptase promoter mutations in hepatocellular carcinomas from different geographical locations. World J. Gastroenterol..

[34] Guichard C (2012). Integrated analysis of somatic mutations and focal copy-number changes identifies key genes and pathways in hepatocellular carcinoma. Nat. Genet..

[35] Sung WK (2012). Genome-wide survey of recurrent HBV integration in hepatocellular carcinoma. Nat. Genet..

[36] Totoki Y (2014). Trans-ancestry mutational landscape of hepatocellular carcinoma genomes. Nat. Genet..

[37] Schulze K (2015). Exome sequencing of hepatocellular carcinomas identifies new mutational signatures and potential therapeutic targets. Nat. Genet..

[38] Lau CC (2014). Viral-human chimeric transcript predisposes risk to liver cancer development and progression. Cancer Cell.

[39] Honeyman JN (2014). Detection of a recurrent DNAJB1-PRKACA chimeric transcript in fibrolamellar hepatocellular carcinoma. Science.

[40] He S (2016). Accuracy of microRNAs for the diagnosis of hepatocellular carcinoma: A systematic review and meta-analysis. Clin. Res. Hepatol. Gastroenterol..

[41] Jiao J (2018). Telomerase reverse transcriptase mutations in plasma DNA in patients with hepatocellular carcinoma or cirrhosis: Prevalence and risk factors. Hepatol. Commun..

[42] Ng CKY (2018). Circulating Cell-Free DNA in Hepatocellular Carcinoma: Current Insights and Outlook. Front. Med..

[43] Tomimaru Y, E.H., Nagano H (2012). Circulating microRNA-21 as a novel biomarker for hepatocellular carcinoma. J. Hepatol..

[44] Little, S. Amplification-refractory mutation system (ARMS) analysis of point mutations. Curr Protoc Hum Genet, Chapter 9: p. Unit 9 8 (2001). 10.1002/0471142905.hg0908s07 18428319

