# Supplementary material for: Clinical significance of combined circulating TERT promoter mutations and miR-122 expression for screening HBV-related hepatocellular carcinoma
Source: Sci Rep. 2020 May 18;10:8181. doi: 10.1038/s41598-020-65213-8 (PMC7234991; doi:10.1038/s41598-020-65213-8)
Supplement: Supplementary file 1 — Supplementary Information. [file 41598_2020_65213_MOESM1_ESM.docx]

**Supplementary materials**

**Title: Clinical significance of combined circulating *TERT* promoter mutations and miR-122 expression for screening HBV-related hepatocellular carcinoma**

Ngo Tat Trung^1,2,*,#^, Nghiem Xuan Hoan^2,3,*^, Pham Quang Trung^1,2^, Mai Thanh Binh^4^, Hoang Van Tong^5,6^, Nguyen Linh Toan^6^, Mai Hong Bang^4^, Le Huu Song^2,3,#^

^(1)^ Centre for Genetic Consultation and Cancer Screening, 108 Military Central Hospital,

^(2)^ Vietnamese-German Center of Excellence in Medical Research, Hanoi, Vietnam

^(3)^ Institute of Clinical Infectious Diseases, 108 Military Central Hospital, Hanoi, Vietnam

^(4)^ Department of Gastroenterology, 108 Military Central Hospital, Hanoi, Vietnam

^(5)^ Institute of Biomedicine and Pharmacy, Vietnam Military Medical University, Hanoi, Vietnam

^(6)^ Department of Pathophysiology, Vietnam Military Medical University, Hanoi, Vietnam

^(*)^ Equally contributed to the work

^(#)^ Corresponding authorship

**Corresponding authors:**

**Dr. Ngo Tat Trung, PhD.**

Centre for Genetic Consultation and Cancer Screening

108 Military Central Hospital

No 1, Tran Hung Dao Street,

Hai Ba Trung Dist, Hanoi, Vietnam

Tel. +84 919119416

Email: [trungnt@benhvien108.vn](mailto:trungnt@benhvien108.vn)

**Assoc. Prof. Dr. Le Huu Song, MD, PhD**.

Institute of Clinical Infectious Diseases,

108 Military Central Hospital

No 1, Tran Hung Dao Street,

Hai Ba Trung Dist, Hanoi, Vietnam

Tel. +84 69 698713/ Fax. +84 439728027

Email: [lehuusong@108-icid.com](mailto:lehuusong@108-icid.com)

**Supplementary figure 1**

**Supplementary figure 1**. Detection limit of nested PCR assays for identification of *TERT* promoter mutations
